# Epithelial-to-mesenchymal transition markers to predict response of Berberine in suppressing lung cancer invasion and metastasis

**DOI:** 10.1186/1479-5876-12-22

**Published:** 2014-01-24

**Authors:** Hui-wei Qi, Ling-yan Xin, Xin Xu, Xian-xiu Ji, Li-hong Fan

**Affiliations:** 1 Department of Oncology, Shanghai Pulmonary Hospital, Tongji University School of Medicine, Shanghai, PR China; 2 School of Medicine, Suzhou University, Suzhou, PR China

**Keywords:** Lung neoplasms, Berberine, Invasiveness, Epithelial-mesenchymal transition, Transforming growth factor beta1

## Abstract

**Background:**

The effects of berberine on the metastatic potential of lung cancer cells and its underlying mechanisms have not been fully elucidated. Since epithelial-to-mesenchymal transition is a cellular process associated with cancer invasion and metastasis, we attempted to investigate the potential use of berberine as an inhibitor of TGF-β1-induced epithelial-to-mesenchymal in A549 cells.

**Methods:**

In this study, we investigated the anticancer activity of berberine against A549 cells *in vitro* and *in vivo*. BBR-induced apoptosis of the human lung cancer cells was determined by flow cytometry. The ability of BBR to inhibit TGF-β-induced EMT was examined by QRT-PCR and Western blotting. The impact of BBR on A549 cell migration and invasion was evaluated by transwell assay.

**Results:**

We demonstrated that TGF-β1 induced epithelial-to-mesenchymal to promote lung cancer invasion and metastasis. Berberine inhibited invasion and migration of A549 cells, increased expression of the epithelial phenotype marker E-cadherin, repressed the expression of the mesenchymal phenotype marker Vimentin, as well as decreased the level of epithelial-to-mesenchymal -inducing transcription factors Snail1 and Slug during the initiation of TGF-β1-induced epithelial-to-mesenchymal. Furthermore, berberine inhibited growth of lung cancer cells *in vivo* xenograft.

**Conclusions:**

Our findings provided new evidence that berberine is an effective inhibitor of the metastatic potential of A549 cells through suppression of TGF-β1-induced epithelial-to-mesenchymal.

## Background

Lung cancer is the leading cause of cancer-related mortality both worldwide and in China [1]. Non-small cell lung cancer (NSCLC) represents nearly 80% of all lung cancers. More than 70% of patients with lung cancer are at advanced stages at diagnosis, and the prognosis of these patients remains poor. Standard therapies such as chemotherapy and radiotherapy have provided only limited improvement in many cases. This dismal clinical and epidemiological picture underscores the need for novel treatment strategies to target this aggressive disease.

TGF-β is a cytokine known to have a biphasic effect on tumor progression. Although TGF-β can function as a tumor suppressor through inhibition of cell proliferation of non-transformed cells, it can also mediate tumor progression by promoting epithelial to mesenchymal transition (EMT) [2-4]. TGF-β-induced EMT is an important step implicated in cell invasion and metastasis in lung cancer [5,6]. EMT, a biologic program seen in several types of epithelial cancers including NSCLC, is associated with increased invasion, migration, and cell proliferation [7-9]. The EMT process consists of several sequential steps: dissolution of cell-cell adhesions, loss of apical-basolateral polarity, reorganization of the actin cytoskeleton, and increases in cell motility.

**Figure 1:**
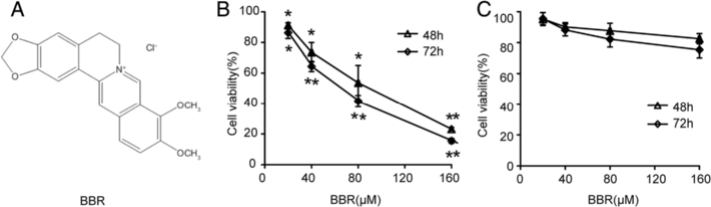
**The effect of BBR on cell viability in A549 cells. (A)** Chemical structure of berberine hydrochloride. A549 cells **(B)** and nomal human bronchial epithelial cells **(C)** were treated with 0, 20, 40, 80 and 160 μM BBR for 48h and 72h. Cell viability was measured using MTT assay. Values are expressed as mean±SD of three experiments. *P<0.05, **P<0.01, as compared with control.

Berberine (2, 3-methylenedioxy-9, 10-dimenthoxyprotoberberine chloride, BBR) (Figure 1A), a clinically important natural isoquinoline alkaloid derived from Berberis species, is characterized by a diversity of pharmacological effects [10-12]. BBR is widely used as an antibacterial, antifungal, and anti-inflammatory drug, and has been used as a gastrointestinal remedy for thousands of years in China [13]. In recent years, anti-cancer activity of BBR has been explored in various types of cancer including lung cancer. The antineoplastic properties of BBR include inhibition of proliferation and induction of apoptosis, along with inhibition of cell migration and invasion via regulation of multiple pathways [14-17]. The potential effects of berberine include DNA topoisomerase inhibition, DNA or RNA binding, NF-kappa B signal activation, mitochondrial function, matrix metalloproteinase regulation, reactive oxygen species generation, and p53 activation [18-21].

However, the underlying molecular mechanisms through which BBR inhibits cell migration and invasion in lung cancer have not been fully elucidated. In this study, we examined the effects of BBR on A549 lung cancer cells, especially the effect on TGF-β-induced EMT which promotes A549 lung cancer cell migration and metastasis. Our results demonstrate that BBR inhibits TGF-β-induced EMT in A549 lung cancer cells.

## Methods

### Reagents and antibodies

BBR was obtained from Sigma and was dissolved at a concentration of 100 mM in dimethyl sulfoxide (DMSO, Sigma-Aldrich) as a stock solution (stored at -20°C). It was then diluted to working concentrations with cell culture medium. The maximum final concentration of DMSO was less than 0.1% for each treatment, and was also used in controls. Recombinant human TGF-β1 was purchased from Peprotech. Rabbit monoclonal antibodies against human E-cadherin, Slug, Snail, Vimentin, MMP-2 and MMP-9 were purchased from Epitomics. P-Smad2/3(Ser423/425) and Smad 2/3 were purchased from Cell Signaling. Matrigel (BD Biosciences) and 24-well transwells (Corning) were used.

### Cell culture and drug treatment

The A549 human NSCLC cell line (American Type Culture Collection) in this study was maintained in Dulbecco’s Modified Eagle’s Medium (DMEM) containing 10% fetal bovine serum (FBS), 100 units/mL penicillin, and 100 mg/mL streptomycin. Cells were incubated in a humidified, 5% CO^2^ atmosphere at 37°C.

### MTT assay for cell viability/proliferation

The effect of BBR on cell viability/proliferation was determined using MTT assay. Briefly, 1 × 10^4^ cells per well were plated in 96-well culture plates. Incubated overnight, the cells were treated with various concentrations of BBR (0, 20, 40, 80 and 160 μM) for 48 h and 72 h. The cells were then treated with 10 μL of 5 mg/mL MTT (Sigma-Aldrich) and incubated for 4 h at 37˚C. The medium was then discarded, and 200 μL of DMSO was added to dissolve the resulting formazan crystals. Absorption values at 490 nm were determined with Multiskan MS microplate reader (Labsystems, Finland). The cell viability of BBR-treated cells was calculated as the percentage of cell viability compared to untreated cells, which were arbitrarily assigned 100% viability.

### Flow cytometric analysis for apoptotic cell death

Flow cytometric analysis was used to determine BBR-induced apoptosis of the human lung cancer cells using the Annexin V-conjugated Alexa Fluor488 (Alexa488) Apoptosis Detection Kit (Invitrogen) following the instructions of the manufacturer. Briefly, after overnight serum starvation, cells were treated with various concentrations of BBR for desired time points. The cells were then harvested, and incubated with Alexa488 and propidium iodide. The stained cells were analyzed by fluorescence-activated cell sorting (FACS) using a FACS Calibur instrument (BD Biosciences) equipped with Cell Quest 3.3 software.

### Quantitative real-time reverse transcription-polymerase chain reaction (QRT-PCR)

Total RNA was extracted using TRIZOL reagent (Invitrogen) as per standard protocol. RNA (1 μg) was used as template for reverse transcription reaction (Takara, Japan), followed by quantitative real-time RT-PCR (QRT-PCR) analysis using specific primers for E-cadherin, Vimentin and GAPDH. Primer sequences were as followed: E-cadherin, forward primer 5’-TGCCCAGAAAATGAAAAAGG-3’, reverse primer 5’-GTGTAYGTGGCAATGCGTTC-3’; Vimentin, forward primer 5’-GAGAACTTTGCCGTTGAAGC-3’, reverse primer 5’-GCTTCCTGTAGGTGGCAATC-3’, GAPDH, forward primer 5’-GAGAGACCCTCACTGCTG-3’, reverse primer 5’-GASTGGTAGATGACAAGGTGC-3’. The samples were assessed by 2 - ΔΔCt relative quantitative analysis to determine the expression differences.

### Protein extraction and Western blot

Cells were lysed and total protein was extracted. Briefly, cells were lysed in buffer containing 50 mM Tris, pH 7.4, 150 mM NaCl, 1 % Triton X-100, 10% glycerol, 5 mM EDTA, 1 mM sodium vanadate, 1 mM glycerophosphate, 1 mM sodium fluoride, 2ug/mL leupeptin, 10 μg/mL aprotinin, and 1 mM phenylmethylsulfonyl fluoride (PMSF). Lysates were collected and centrifuged at 4°C at 12000 r/min for 20 min to pellet cell debris. Protein concentration was quantified by BCA protein assay. A total of 60 μg of protein was added to loading buffer, heated at 100°C for 5 min, separated on 10% polyacrylamide gel and transferred to nitro-cellulose membranes. The membranes were blocked in 5% non-fat milk in TBST buffer (Tris Buffer Saline containing 0.1% Tween-20) for 1 h at room temperature, and incubated overnight by the appropriately diluted primary antibodies at 4°C. After extensive washing with TBST buffer, the blots were incubated with HRP-conjugated secondary antibody for 1 h at room temperature. After extensive washing with TBST buffer, target proteins were detected by enhanced chemiluminescence reagents ECL.

### Transwell assay

For transwell migration and invasion assay, about 2.5 × 10^4^ cells cultured in 200 μL medium with 1% fetal bovine serum were plated in the upper chamber of a non-coated transwell insert. In the lower chamber, 600 μL medium with 10% fetal bovine serum was used as a chemo-attractant to encourage cell migration. For the Matrigel invasion assay, the upper chamber of the transwell inserts were coated with 50 μL of 2.0 mg/mL Matrigel, and about 5 × 10^4^ cells were plated in the upper chamber of the Matrigel-coated transwell insert. Cells of both assays were incubated for 24 h and those cells that did not migrate or invade were removed using a cotton swab. All cells were stained using crystal violet staining and counted under a light microscope. We selected four random views to count the cells and each experiment was repeated independently three times.

### Anti-tumor activity of BBR *in vivo* xenograft

Six-week-old male BALB/c athymic nude mice were purchased from Shanghai SLAC Laboratory Animal Co., Ltd (Shanghai, China). A549 cells were injected subcutaneously (2 × 10^6^ cells/0.1 mL PBS/animals) by a 27-gauge needle into the right lower flanks of the mice. After 24 h, the mice were randomly divided in three groups (n = 6), the tumor bearing nude mice were intraperitoneally injected with BBR (5 and 10 mg/kg, three times per week for 40 days), while the control mice received an equal volume of PBS. The weight and tumor volume of the animals were monitored at an interval of 3–4 days. The tumor volumes were measured with vernier calipers and were calculated by the following formula: (A × B^2^)/2, where A was the larger and B was the smaller of the 2 dimensions of the tumor. At the end of the experiment, the animals were sacrificed with cervical dislocation. The tumors were separated from the surrounding muscles and dermis, excised and weighed. This study was carried out in strict accordance with the recommendations in the Guide for the Care and Use of Laboratory Animals of the National Institutes of Health. The protocol was approved by the Committee on the Ethics of Animal Experiments of Tongji University (Permit Number: 12ZR1425900).

### Statistical analysis

Quantitative values were presented as means ± SD. The one-way ANOVA analysis followed by a Tukey-Kramer multiple comparisons test was conducted to compare the corresponding data. Differences with *P* < 0.05 were considered statistically significant.

## Results

### BBR inhibits proliferation of A549 lung cancer cells *in vitro*

First, we determined the cytotoxic effect of BBR on A549 lung cancer cells using an MTT assay. As shown in Figure 1B, A549 cells were treated with various concentrations of BBR (0, 20, 40, 80 and 160 μM) for 48 h and 72 h. It was observed that BBR inhibited proliferation of A549 cells in a dose- and time-dependent manner (*P* < 0.05). After 72 h of BBR (80 μM) treatment, cell viability was reduced by approximately 60%. IC50 value for BBR in A549 cells was 56.15 ± 3.14 μM. We also examined the effect of BBR on normal human bronchial epithelial cells. In contrast, no marked cytotoxic effects were seen in normal human bronchial epithelial cells when exposed to the same concentrations of BBR for 48 h and 72 h (Figure 1C).

### BBR induces apoptosis of A549 lung cancer cells *in vitro*

**Figure 2:**
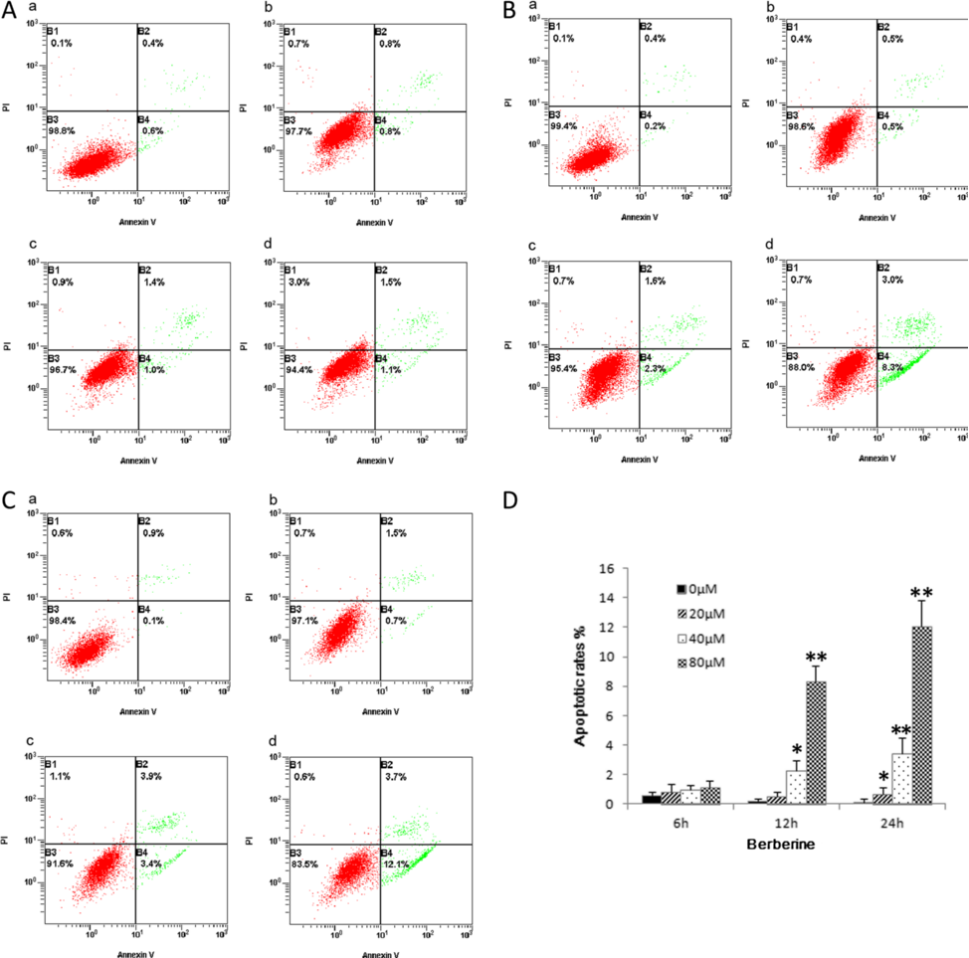
**Effect of BBR on cell apoptosis in A549 cells.** A549 cells were treated with 0, 20, 40 and 80 μM BBR for 6 h **(A)**, 12 h **(B)** and 24 h **(C)**. Apoptotic rates were measured using flow cytometry **(D)**. Values are expressed as mean ± SD of three experiments. *P <0.05, **P < 0.01, as compared with control.

To examine whether BBR-induced inhibition of cell proliferation of A549 lung cancer cells was associated with the induction of apoptosis, we analyzed the apoptotic rates of A549 cells in the treatment of BBR by flow cytometry. A549 cells were treated with various concentrations of BBR (0, 20, 40 and 80 μM) for 6 h, 12 h and 24 h, respectively. It can be seen in Figure 2 that A549 cells displayed apoptotic features after treatment with BBR for 12 h and 24 h. BBR induced cell apoptosis of A549 cells in a dose- and time-dependent manner (*P* < 0.05).

### BBR inhibits morphological changes of TGF-β1-induced EMT

**Figure 3:**
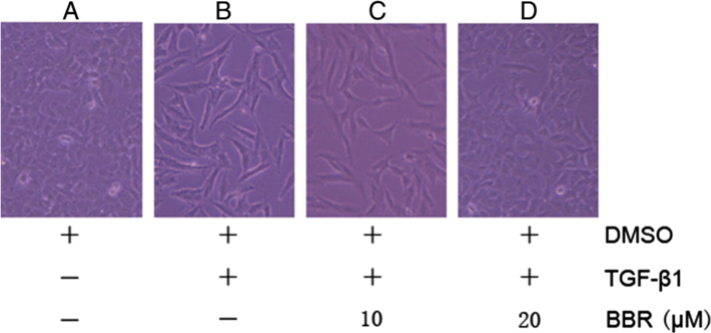
**BBR inhibits TGF- β1-induced EMT development. (A)** Control A549 cells treated with DMSO display classical epithelial morphology (control group); A549 cells show a pebble-like shape and tight cell-cell adhesion. **(B)** Morphological changes of A549 cells induced by 5 ng/mL of TGF-β1 for 48 h; TGF-β1-treated cells show a decrease in cell-cell contacts and adopt a more elongated morphological shape representing a mesenchymal phenotype (TGF-β group). **(C)** A549 cells treated with 5 ng/mL of TGF-β1 plus 10 μM of BBR for 48 h also show the mesenchymal phenotype (TGF-β + BBR 10 group). **(D)** A549 cells treated with 5 ng/mL of TGF-β1 plus 20 μM of BBR for 48 h display epithelial morphology (TGF-β + BBR 20 group) (Magnification × 200).

We sought to determine whether BBR could inhibit TGF-β1-induced EMT. A549 lung cancer cells were used for this study because we have induced EMT in A549 lung cancer cells via the use of TGF-β1. A549 cells were treated with 5 ng/mL TGF-β1 and then with 0, 5, 10 and 20 μM of BBR respectively for 48 h. A549 cells showed a mesenchymal phenotype after treatment with TGF-β1, but after adding BBR, the cells changed back to epithelial morphology (Figure 3). These findings indicate that BBR could inhibit the effects of TGF-β1 on EMT.

### BBR regulates EMT marker expression duringTGF-β-induced EMT

**Figure 4:**
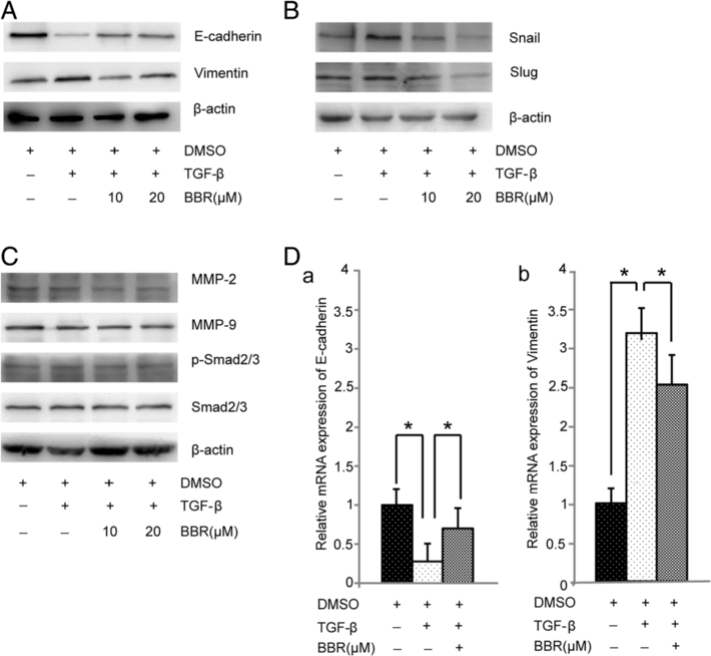
**BBR inhibits TGF-β1-induced EMT markers and transcription factors. (A)** Protein level of E-cadherin was measured by Western blotting assay after A549 cells were treated with different concentrations of BBR for 48 h. **(B)** Protein level of Snail1and Slug were measured by Western blotting assay after A549 cells were treated with different concentration of BBR for 48 h. **(C)** Protein level of MMP2, MMP-9, p-Smad2/3 and Smad2/3 were measured by Western blotting assay after A549 cells were treated with different concentration of BBR for 48 h. **(D)** mRNA level of E-cadherin and Vimentin was measured by real-time PCR assay after A549 cells were treated with 20 μM BBR for 24 h. Values are expressed as mean ± SD of three experiments.*P <0.05, **P <0.01, as compared with control group or TGF-β1 stimulation group.

To examine whether BBR inhibit TGF-β-induced EMT, A549 cells were treated with DMSO (control group), 5 ng/mL TGF-β1 (TGF-β group), or 5 ng/mL TGF-β1 plus 20 μM BBR (TGF-β + BBR group), and the expression levels of E-cadherin and Vimentin were measured using QRT-PCR and Western blotting. As shown in Figure 4D, compared with control group, TGF-β1 down-regulated the expression of epithelial phenotype marker E-cadherin (*P* < 0.05) and up-regulated the expression of mesenchymal phenotype marker Vimentin (*P* < 0.05). After treatment with BBR, the expression level of E-cadherin increased, while that of Vimentin decreased significantly. Western blotting analysis also demonstrated that BBR released the inhibition of E-cadherin by TGF-β1 and blocked the activation of Vimentin induced by TGF-β1 (Figure 4A).

### BBR represses expressions of EMT-induced transcription factors

To examine the ability of BBR to repress expression of EMT-induced transcription factors, the expression levels of Snail1 and Slug were measured using QRT-PCR and Western blotting. The results showed that Snail1 and Slug were significantly increased in the TGF-β group compared with the control group, and BBR inhibited TGF-β-induced Snail1 and Slug levels in A549 cells (Figure 4B).

### Berberine inhibits the expression of TGF-β1-induced MMP-2, but not MMP-9

Over-expression of MMPS is related to tumor invasion and metastasis. In this experiment, Western blotting was performed to investigate the effects of BBR on the regulation of the expression of MM-2 and MMP-9 in A549 cells. Compared with the control group, the expression of MMP-2 was up-regulated by TGF-β1 but was reversed by treatment with BBR (Figure 4C). The expression of MMP-9 had no change before and after the treatment. Considering that TGF-β/Smad signaling pathway is a classical pathway triggered by phosphorylation of the Smad2/Smad3 [22], we also examined the effects of BBR on the regulation of the Smad2/3 expression. Our results showed that the expression of p-Smad2/3 was down-regulated by BBR in a dose-dependent manner (Figure 4C).

### BBR inhibits TGF-β1-induced migration and invasion in A549 cells

**Figure 5:**
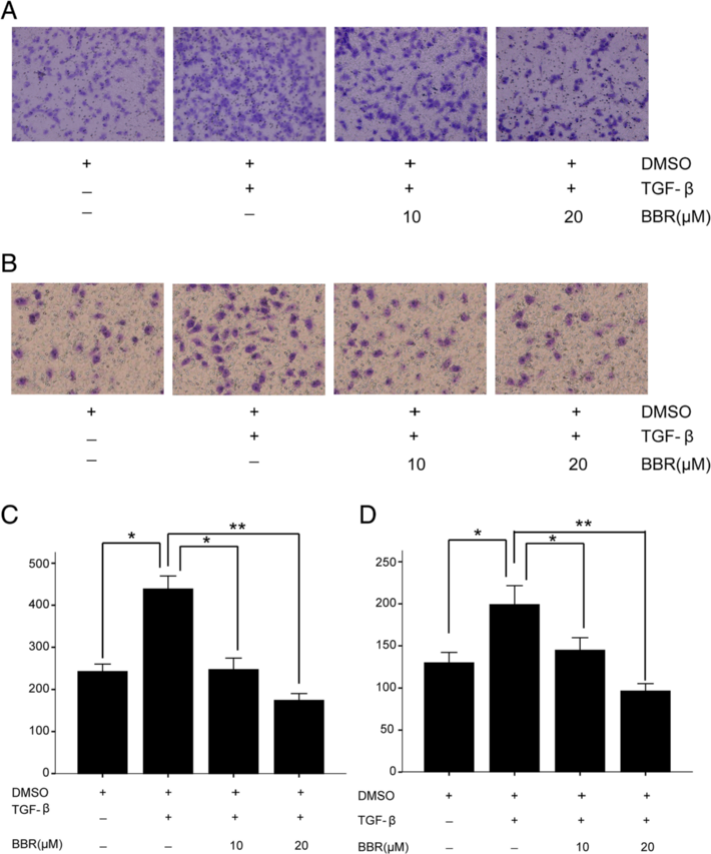
**Effect of BBR on lung cancer cell migration and invasion induced by TGF-β1. (A)** Effects of BBR on lung cancer cell migration after crystal violet staining by Matrigel migration assay as described (100×). **(B)** Effects of BBR on lung cancer invasion after crystal violet staining by Matrigel migration assay as described (100×). **(C)** Matrigel migration of A549 cells counted in five random views. **(D)** Matrigel invasion of A549 cells counted in five random views. Three independent experiments were performed, *P < 0.05, **P < 0.01.

In order to confirm whether BBR affects the process of A549 cell metastasis and invasion after stimulation by TGF-β1, A549 cells were treated with DMSO (control group), 5 ng/mL TGF-β1 (TGF-β group), 5 ng/mL TGF-β1 plus 10 μM BBR (TGF-β + BBR10 group), or 5 ng/mL TGF-β1 plus 20 μM BBR (TGF-β + BBR20 group), and transwell assay was used to determine the impact of BBR on A549 cell migration and invasion. In terms of migration and invasion, a significant difference was observed between the control group and TGF-β group (*P* < 0.05) (Figure 5). This result showed that TGF-β1 can promote lung cancer cell metastasis. We also found that BBR inhibited A549 cell metastasis induced by TGF-β in a dose-dependent manner, and the difference between the TGF-β group and TGF-β + BBR10 or TGF-β + BBR20 group was significant (*P* < 0.05).

### BBR inhibits growth of lung cancer cells *in vivo* xenograft

**Figure 6:**
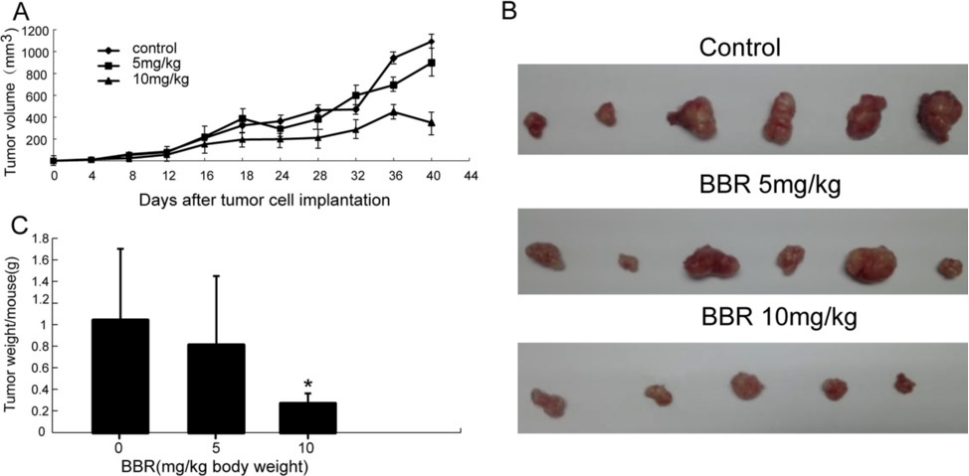
**Inhibition of tumor growth in nude mice xenografted with human A549 cells by BBR.** Mice were randomly divided into three groups, five to six mice each. In treated mice, BBR was administered i.p. at a dose of 5 mg/kg or 10 mg/kg three times per week. A significant reduction in tumor volume was observed in treated mice. The figures show the relative tumor volume **(A, B)** and tumor weight **(C)**.

We have observed that treatment of A549 cells *in vitro* with BBR induces apoptosis. The body weight and hair coats, as well as other overall behavioral activities were similar in the all groups at the completion of the experiments, suggesting that BBR did not have major side effects on these mice (data not shown). Tumor volume was measured three times per week, and all mice were sacrificed at the end of 40 days when tumors were dissected and weighted. As shown in Figure 6A, tumor volume was 1.04 ± 0.66 cm^3^ in control group, 0.81 ± 0.64 cm^3^ in mice administered BBR at a concentration of 5 mg/kg body weight and 0.27 ± 0.10 cm^3^ in mice administered BBR at a concentration of 10 mg/kg body weight, respectively. The wet weight tumor/mouse ratio was also recorded. As shown in Figure 6C, the relative wet weight of the A549 tumors was 23% (not significant) and 71% lower (*P* < 0.05) in mice treated with 5 mg BBR/kg body weight and 10 mg BBR/kg body weight, respectively, as compared with the control group.

## Discussion

Many plant-derived agents with few adverse effects have been accepted as potential alternatives to the therapy for lung cancer. BBR is an isoquinoline alkaloid that has long been used as a stomachic, an antidiarrheal agent, an antibiotic and an anti-inflammatory in Asian countries and has been shown to have few side effects [23-25]. BBR has been reported to affect various biological functions, including cell cycle progression, cell apoptosis and growth. The mechanism of its antitumor activity differs among cancer cell lines [26-29].

In this study, the data clearly demonstrated that BBR inhibited cell proliferation and induced cell apoptosis of A549 in a dose- and time-dependent manner (*P* < 0.05) (Figures 1 and 2). After treatment with BBR in lung cancer xenograft-bearing nude mice, we found that intraperitoneal administration of BBR at a dosage of 10 mg/kg caused a significant decline in tumor volume and weight of (*P* < 0.05) (Figure 6). These all demonstrated that BBR can inhibit A549 cell proliferation in vitro and in vivo. In contrast, such cytotoxicity of BBR in A549 lung cancer cells was not discovered in normal human bronchial epithelial cells, indicating a high specificity against malignant cell and a plausible explanation for its few side effects. The differential cytotoxic effects of BBR on malignant and normal cells were also reported to exist in hepatoma cells [12] and prostate cancer [19].

Recent studies have revealed the potential therapeutic effect of BBR against invasion and metastasis of various cancer cell lines. BBR inhibits melanoma cell invasion and metastasis by inhibition of COX-2, PGE2 and PGE2 receptors [17] and several other signaling molecules such as ERK1/2, NF-κB, ATF-2 and CREB which are involved in the transcriptional regulation of matrix metalloproteinase (MMP) gene expression [30]. Berberine also exerted anti-invasive effect on HepG2 cells through suppression of MMP-9 expression [12]. In the present study, we attempted to observe the involvement of a previously unknown mechanism, EMT, in the BBR-induced suppressive effect on A549 cell invasion and migration.

Cancer metastasis is a complex, multi-step, and continuous process that includes proliferation, migration, invasion, adhesion and angiogenesis. EMT is characterized by the loss of cell-cell adhesion and the increase in cell motility, and it is a key process in cancer progression and metastasis [4], making EMT inhibition an attractive therapeutic strategy. The EMT process is triggered by transcription factors (Twist1, Zeb2, Snail1 and Snail2), growth factors (TGF-β and EGF), inflammatory cytokines (IL-6 and TNF-α), chemokines (SDF-1 and IL-8), and other enzymes or proteins (ID1, PRL3 and Bmi-1) [31-33]. Our previous studies demonstrated that TGF-β1-induced A549 cells undergo morphological alterations characteristic of EMT, including increased metastasis and invasion, up-regulated expression of mesenchymal markers Vimentin and down-regulated expression of E-cadherin epithelial markers [34]. TGF-β1 also enhances expression of zinc-finger transcriptional factors Snail and Slug, which repress E-cadherin transcription. These transcriptional repressors of E-cadherin are required during EMT development [4]. The results of this study showed that BBR decreased A549 cell migration and invasion in a dose-dependent manner and inhibited TGF-β1-induced EMT in A549 cells, as proved by the increase of the expression of the epithelial phenotype marker E-cadherin and the decrease of the mesenchymal phenotype marker Vimentin (Figure 4). Transcriptional factors of Snail1 and Slug play a central role in EMT. Snail1 transcriptional factor binds to the promoter E-box, which represses E-cadherin transcription. During EMT development, TGF-β induced Snail1 expression [4]. In addition, our results demonstrated that expression of EMT inducing transcription factors, Snail1 and Slug, were also inhibited by BBR (Figure 4B). Moreover, EMT is able to increase cell adhesion, migration and invasion in cancer cells [35,36]. Therefore, BBR may inhibit lung cancer cell invasion and metastasis by suppressing TGF-β1-induced EMT.

Although EMT in embryonic development is a coordinated, organized process involving interaction between different cells and tissue types, aspects of the EMT program can be inappropriately activated in response to microenvironmental alterations and aberrant stimuli, and this can contribute to diseased conditions including cancer progression. Specifically, it could be activated in pathologic conditions-especially by matrix metalloproteinases (MMPs) [37]. MMPs differentially expressed by tumor cells and stromal cells play a pivotal role in the degradation of the extracellular matrix (ECM). In this process, cleavage of some ECM components unmasks cryptic sites, generating fragments with new biological activities modulating migration, growth, or angiogenesis. Therefore, up-regulation of MMPs provides clues for tumor metastasis such as tumor-induced angiogenesis, tumor invasion and establishment of metastatic foci at the secondary site [38,39]. Expression analysis of lung cancer cells also demonstrated that BBR treatment significantly down-regulated MMP. In addition to transcription factors, cell signaling molecules are also critical inducers of EMT in the context of development and in cancer. TGF-β/Smad signaling pathway is a classical pathway. In this system, TGF-β1 regulates cellular processes by binding and phosphorylating cell-surface receptors (TGF-βRI/TGF-βRII), the activated TGF-βRI phosphorylates Smad2 or Smad3, which then binds to Smad4. The resulting Smad complex then moves into the nucleus, where it interacts in a cell-specific manner with various transcription factors to regulate the transcription of many genes [22,40].

## Conclusions

In summary, our study provides evidence that BBR inhibits lung cancer cell proliferation in vitro and in vivo, and that BBR may suppress lung cancer cell invasion and metastasis through inhibiting TGF-β1-induced EMT.

## Competing interests

The authors declare that they have no competing interests.

## Authors’ contributions

LYX, XXJ and XX participated in the experiments. HWQ participated in the design of the study, performed the statistical analysis, and drafted the manuscript. LHF conceived of the study, and participated in its design and helped to draft the manuscript. All authors read and approved the final manuscript.
